# Patient Satisfaction With Auditable Pharmaceutical Transactions and Services: A Cross‐Sectional Study at Bule Hora University Teaching Hospital, Ethiopia

**DOI:** 10.1002/hsr2.72657

**Published:** 2026-06-15

**Authors:** Aliyi Gerina, Abduba Wariyo Guyo, Woyesa Elema

**Affiliations:** ^1^ Department of Pharmacy, Institute of Health Bule Hora University Bule Hora Ethiopia

**Keywords:** APTS, Ethiopia, hospital pharmacy, patient satisfaction, pharmaceutical services

## Abstract

**Background and Aims:**

Auditable Pharmaceutical Transactions and Services (APTS) is a national initiative implemented in Ethiopia to improve the transparency, efficiency, and accountability of pharmaceutical services. Although APTS has been widely adopted, its impact on patient satisfaction remains underexplored, especially in resource‐limited settings. This study aimed to assess patient satisfaction with pharmaceutical services delivered under the APTS system by Outpatient Pharmacy of Bule Hora University Teaching Hospital (BHUTH), Ethiopia.

**Methods:**

A hospital‐based cross‐sectional study was conducted among 326 adult outpatients receiving pharmacy services at BHUTH from January 2024 to March 2024. A structured, interviewer‐ questionnaire administered and analyzed using Statistical Package for Social Science (SPSS) version 27.0. Descriptive statistics summarized patient responses with frequency, percentages and 95% confidence intervals (CIs). Associations between socio‐demographic variables and satisfaction were evaluated using *χ* ^2^ tests, with statistical significance defined as *p* < 0.05.

**Results:**

The study included 326 outpatients, with a mean age of 38 ± 14 years; the majority were male (197, 60.4%). Overall, 89.0% (95% CI: 85.8–92.2) of patients reported satisfaction with pharmacy services. Most of the patients were highly satisfied with the physical environment (95.0%), interpersonal skills (93.10%) and service efficiency (96.75%). However, lower satisfaction was noted in medication counseling (75.0%) and medicine access (85.75%). Urban residence was significantly associated with higher satisfaction (*χ* ^2^ = 7.85, *p* = 0.005).

**Conclusion:**

The implementation of APTS at BHUTH resulted in high overall patient satisfaction. Nevertheless, improvements are still needed in medication counseling and medicine supply management. Strengthening pharmacist training, improving counseling practices, and enhancing pharmaceutical supply chain systems may further improve patient satisfaction and the quality of pharmacy services.

## 1 Introduction

The delivery of high‐quality pharmaceutical services is a cornerstone of effective healthcare systems. As the final interface between patients and medicines, pharmacy services play a critical role in ensuring medication safety, adherence, and therapeutic outcomes [1]. Patient satisfaction is widely regarded as a key performance indicator in healthcare and reflects not only the technical quality of services but also the interpersonal aspects of care, such as communication, respect, and accessibility [2].

In Ethiopia, the pharmaceutical sector has long been challenged by issues including inefficient drug supply chains, poor dispensing practices, and a lack of accountability in financial transactions [3]. To address these systemic shortcomings, the Federal Ministry of Health launched the Auditable Pharmaceutical Transactions and Services (APTS) program. APTS is an innovative, evidence‐based intervention aimed at improving the transparency, efficiency, and accountability of pharmaceutical services in public health facilities [4]. It encompasses a standardized set of practices, including automated inventory management, rational drug use, ethical billing, and performance monitoring, while also enhancing the patient experience at pharmacy counters [5].

Several studies have acknowledged the positive impact of APTS on reducing wastage, improving documentation, and increasing revenue retention in public hospitals [4, 6]. However, the extent to which these systemic improvements translate into better patient satisfaction remains underexplored, especially in rural and resource‐limited regions where pharmacy infrastructure and workforce capacity may vary significantly [7].

Patient satisfaction in the context of pharmaceutical services is influenced by a range of factors such as the physical condition of the pharmacy, availability of prescribed medications, waiting time, and the quality of pharmacist‐patient interactions [8]. In particular, effective communication about dosage, potential side effects, and storage instructions is essential for ensuring safe and effective use of medications. Inadequate counseling may lead to misuse, non‐adherence, and reduced confidence in healthcare services [9].

This study was therefore conducted to assess patient satisfaction with pharmaceutical services provided under the APTS system at Bule Hora University Teaching Hospital (BHUTH), one of the major healthcare institutions in southern Ethiopia. Understanding the patient perspective is critical for evaluating the effectiveness of APTS and identifying areas for quality improvement in pharmacy practice.

## 2 Methods

### 2.1 Study Design, Period, and Setting

A hospital‐based cross‐sectional study was conducted over 3 months, from January 1 to March 31, 2024, at the outpatient Pharmacy to assess patient satisfaction with pharmaceutical services provided under the Auditable Pharmaceutical Transactions and Services (APTS) system at Bule Hora University Teaching Hospital (BHUTH), located in West Guji Zone, Oromia Region, Ethiopia. BHUTH serves a large population in southern Ethiopia and implements the APTS framework as mandated by the Ethiopian Federal Ministry of Health.

### 2.2 Study Population

#### 2.2.1 Source Population

All patients who visited the outpatient pharmacy of BHUTH between January 1, 2024, and March 31, 2024.

#### 2.2.2 Study Population

Patients receiving pharmacy services during the study period who met the inclusion criteria.

### 2.3 Eligibility Criteria

#### 2.3.1 Inclusion Criteria

- All adult patients (aged ≥ 18 years) who visited the outpatient pharmacy of BHUTH during the data collection period.
- Patients who had received at least one prescription service from the pharmacy and were willing to participate.
- Caregivers responding on behalf of patients unable to participate independently

#### 2.3.2 Exclusion Criteria

- Patients in critical condition or unable to provide informed responses during the interview process

### 2.4 Sample Size Determination

The sample size was calculated using the single population proportion formula:

$$n=(Z^{2}\times p(1-p))/d^{2}$$

where:

*n *= required sample size

*Z* = 1.96 for 95% confidence interval

*p* = 0.744 (The level of patient satisfaction was taken from previously done research studies with systems improved Access to pharmaceutical services (SIAPS) funded by (USAID) in 2016 Debra Markos Hospital).

*d* = 0.05 (margin of error)

$$n={\left(1.96\right)}^{2}{\;\times \;0.744(1-0.744)/(0.05)}^{2}=296$$

After accounting for 10% non‐response, the final sample size was 326 participants.

### 2.5 Sampling Procedure

A systematic random sampling technique was employed, whereby every *k* ^th^ patient exiting the outpatient pharmacy was selected for the interview, using the daily pharmacy register as the sampling frame. The total outpatient pharmacy visits (16,672) used as the sampling frame were obtained retrospectively from pharmacy records covering the preceding 3 months. The sampling interval (*k*) was calculated as: *k* = 16,672/326 ≈ 50. Every 50th patient was selected, with the first respondent chosen through simple random sampling. Based on the average daily patient flow (approximately 185 patients/day), approximately 3–4 patients were selected per day using systematic random sampling.

### 2.6 Data Collection Tool

Data were collected using a structured, interviewer‐administered questionnaire adapted from previously validated patient satisfaction instruments [10] and aligned with the Ethiopian Ministry of Health APTS checklist. Patient satisfaction with pharmaceutical services delivered under the APTS system was assessed using structured Likert‐scale questionnaire items across multiple service domains. The questionnaire covered five domains:

1. Physical environment: location, counter convenience, cleanliness and comfort of dispensing area
2. Medication counseling: clarification of medication use instruction, explanation of medication side effects, storage instructions, and treatment expectations
3. Interpersonal skills: time spent with patients, courtesy and respect, listening to and answering patients' questions, and privacy during consultations
4. Service efficiency: waiting time and promptness
5. Medicine access: availability of prescribed medicines and fairness of medication costs.

A pretest was conducted on 5% of the sample at a nearby hospital. Minor adjustments were made to ensure clarity, cultural relevance, and appropriateness.

### 2.7 Data Quality Assurance

To ensure data quality, data collectors and supervisors received 1 day of training on the purpose of the study, ethical considerations, and proper interviewing techniques. The principal investigator monitored data collection daily to address inconsistencies or omissions promptly. Completed questionnaires were checked for completeness and accuracy before data entry.

### 2.8 Data Analysis

Data were coded and entered into Microsoft Excel 2019, then exported to SPSS version 27 (IBM Corp., Armonk, NY, USA) for analysis. Descriptive statistics: frequencies, percentages, and means ± SDs summarized demographic characteristics and satisfaction scores. Proportion calculations: satisfaction percentages reported with 95% confidence intervals (CIs). Inferential statistics: *χ* ^2^ tests evaluated associations between patient characteristics and overall satisfaction. Statistical significance was defined as *p* < 0.05, with all tests two‐sided.

### 2.9 Operational Definitions

Auditable Pharmaceutical Transactions and Services (APTS): a system reform approach implemented in Ethiopian public hospitals to improve transparency, accountability, and quality of pharmaceutical services [11].

Satisfied: refers to respondents scoring ≥ 3 on the five‐point Likert scale [11].

Dissatisfied: refers to respondents scoring < 3 on the same scale [11].

### 2.10 Ethical Considerations

This study was reviewed and approved by the Institutional Review Board (IRB) of Bule Hora University, Institute of Health (Ref. No: BHU/IoH/IRB/0426/075/2023). Written informed consent was obtained from all participants prior to data collection. Participants were fully informed about the study's purpose, procedures, confidentiality safeguards, and their right to withdraw at any time without penalty. All study procedures adhered to the ethical principles outlined in the Declaration of Helsinki.

## 3 Results

### 3.1 Demographic Characteristics of the Study Participants

The socio‐demographic characteristics of the study participants are presented in Table 1. The study included 326 outpatients, with a mean age of 38 ± 14 years; the majority were male (197, 60.4%). Regarding age distribution, the largest proportion of participants was in the 26–40 years age group 136 (41.7%), followed by those aged ≥ 41 years 114 (34.7%), while 76 (23.3%) were aged 18–25 years.

**Table 1:** Demographic characteristics of study participants of the outpatient pharmacy of Bule Hora University Teaching Hospital, Ethiopia, 2024 (*n* = 326).

Demographic variable	Category	Frequency (*n*)	Percentage (%)
Gender	Male	197	60.4
	Female	129	39.6
Age group	18–25 years	76	23.3
	26–40 years	136	41.7
	≥ 41 years	114	34.7
Marital status	Married	258	79.1
	Single	68	20.9
Residence	Urban	115	35.3
	Rural	211	64.7
Educational status	Primary school and less	189	57.98
	Secondary school and above	137	40.02
Employment	Farmer	91	27.9
	Housewife	84	25.8
	Government employee	46	14.1
	Merchant	33	10.1
	Other	72	22.1
Type of visit	New	214	65.6
	Repeat	112	34.4
Payment mode	Cash	178	54.6
	Credit	148	45.4

With respect to marital status, the majority of respondents were married 258 (79.1%), whereas 68 (20.9%) were single. In terms of residence, a larger proportion of participants were rural residents 211 (64.7%), while 115 (35.3%) were from urban areas. Regarding educational status, 189 (57.98%) of respondents had completed primary education, while 137 (40.02%) had completed secondary school and above.

Occupationally, farmers constituted the largest group 91 (27.9%), followed by housewives 84 (25.8%), government employees 46 (14.1%), and merchants 33 (10.1%), while 72 (22.1%) were categorized under other occupations. Regarding healthcare service utilization, the majority of participants were new visitors to the pharmacy 214 (65.6%), whereas 112 (34.4%) were repeat visitors. Concerning payment mode, 178 (54.6%) of respondents paid for services in cash, while 148 (45.4%) used insurance (Table 1).

### 3.2 Patient Satisfaction With Pharmaceutical Services

**Figure 1:**
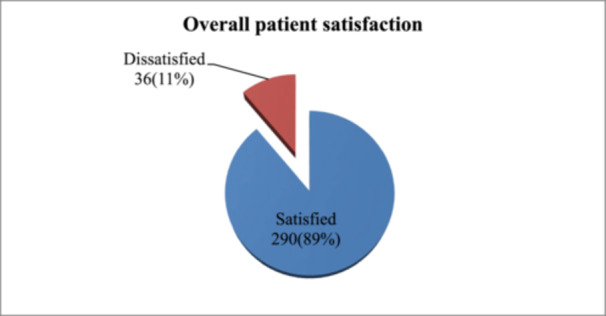
Overall patient satisfaction with pharmaceutical services provided by the outpatient pharmacy of Bule Hora University Teaching Hospital, Ethiopia, 2024 (*N* = 326).

Patient satisfaction with pharmacy services under APTS was 290 (89.0%; 95% CI: 85.8–92.2) participants (Figure 1).

**Figure 2:**
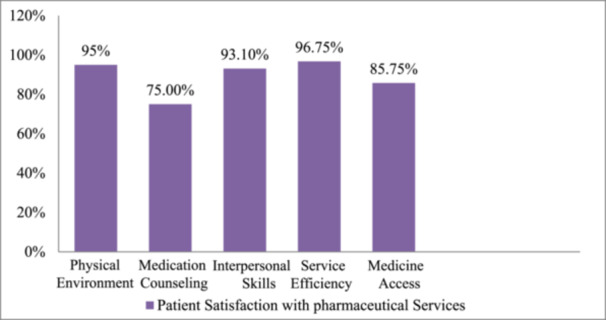
Patient satisfaction with pharmaceutical services provided by the outpatient pharmacy of Bule Hora University Teaching Hospital, Ethiopia, 2024 (*N* = 326).

Patient satisfaction with pharmacy services was assessed across five broad domains: Most participants were highly satisfied with physical environment (95.0%), interpersonal skills (93.10%), and service efficiency (96.75%); although lower satisfaction was noted in medication counseling (75.0%) and medicine access (85.75%) (Figure 2).

In the physical environment domain, satisfaction was generally high. The majority of participants reported satisfaction with the location of the pharmacy 295 (90.5%; 95% CI: 87.3–93.7), the cleanliness and comfort of the dispensing area 314 (96.3%; 95% CI: 94.3–98.3), and the convenience of the dispensing counter 320 (98.2%; 95% CI: 96.8–99.6). These results suggest that the physical layout and organization of the pharmacy contributed positively to the patient experience.

Satisfaction with medication counseling was moderate. Most participants were satisfied with the clarity of medication instructions 279 (85.6%; 95% CI: 81.8–89.4). However, lower satisfaction was observed regarding information on medication storage 230 (70.6%; 95% CI: 65.7–75.5), treatment expectations 243 (74.5%; 95% CI: 69.8–79.2), and explanation of potential side effects 226 (69.3%; 95% CI: 64.3–74.3). This indicates gaps in comprehensive counseling practices.

High levels of satisfaction were reported for interpersonal skills. Participants expressed satisfaction with pharmacists' ability to listen and answer questions 275 (84.4%; 95% CI: 80.5–88.3), time spent with patients 310 (95.1%; 95% CI: 92.8‐97.4), demonstrate courtesy and respect 318 (97.5%; 95% CI: 95.8–99.2), and maintain privacy during consultations 311 (95.4%; 95% CI: 93.1–97.7). These findings highlight the importance of respectful, patient‐centered interactions in pharmacy services.

Satisfaction with service efficiency indicators was very high. Most participants reported satisfaction with the promptness of service 317 (97.2%; 95% CI: 95.4–99.0), and waiting times 314 (96.3%; 95% CI: 94.3–98.3). These results indicate effective workflow and minimal delays during patient service delivery.

In the medicine access domain, satisfaction varied. While the fairness of medication costs 321 (98.5%; 95% CI: 97.2–99.8) was highly rated, fewer participants were satisfied with the availability of prescribed medicines 238 (73.0%; 95% CI: 68.2–77.8). This suggests that supply chain and stock management remain areas requiring improvement (Table 2).

**Table 2:** Patient satisfaction with pharmaceutical services provided by the outpatient pharmacy of Bule Hora University Teaching Hospital, Ethiopia, 2024 (*n* = 326).

Domain	Indicators	Satisfied *n* (%)	95% Confidence interval
Physical environment	Location of the pharmacy	295 (90.5%)	87.3–93.7
	Cleanliness and comfort of the dispensing area	314 (96.3%)	94.3–98.3
	Counter convenience	320 (98.2%)	96.8–99.6
Medication counseling	Clarity of medication instructions	279 (85.6%)	81.8–89.4
	Storage information	230 (70.6%)	65.7–75.5
	Treatment expectations	243 (74.5%)	69.8–79.2
	Explanation of side effects	226 (69.3%)	64.3–74.3
Interpersonal skills	Listening and answering of patient's questions	275 (84.4%)	80.5–88.3
	Time spent with patients	310 (95.1%)	92.8–97.4
	Courtesy and respect	318 (97.5%)	95.8–99.2
	Privacy during consultation	311 (95.4%)	93.1–97.7
Service efficiency	Promptness of pharmacy service	317 (97.2%)	95.4–99.0
	Waiting time	314 (96.3%)	94.3–98.3
Medicine access	Availability of prescribed medicines	238 (73.0%)	68.2–77.8
	Fairness of the cost of medicine	321 (98.5%)	97.2–99.8

### 3.3 Factors Associated With Overall Patient Satisfaction

The association between selected patient characteristics and overall satisfaction with pharmacy services is presented in Table 3. Among the variables examined, place of residence showed a statistically significant association with patient satisfaction (*χ* ^2^ = 7.85, *p* = 0.005). Patients residing in urban areas reported a higher level of satisfaction 110/115 (95.7%), compared with those from rural areas 180/211 (85.3%). This finding suggests that geographical differences may influence patients' perceptions of pharmacy services, possibly due to variations in access to health information, healthcare expectations, or familiarity with pharmaceutical services.

**Table 3:** Association between patient characteristics and overall satisfaction of pharmaceutical service provided by outpatient pharmacy of Bule Hora University Teaching Hospital, Ethiopia, 2024 (*n* = 326).

Variable	Category	Satisfied (*n*) (%)	*χ* ^2^	*p* value
Gender	Male	177/197 (89.8)	0.12	0.73
	Female	113/129 (87.6)		
Age group	18–25 years	67/76 (88.2)	0.43	0.81
	26–40 years	120/136 (88.2)		
	≥ 41 years	103/114 (90.4)		
Marital status	Married	230/258 (89.1)	0.02	0.88
	Single	60/68 (88.2)		
Residence	Urban	110/115 (95.7)	7.85	0.005*
	Rural	180/211 (85.3)		
Educational status	Primary school and less	172/189 (91.0)	5.12	0.08
	Secondary school and above	118/137 (86.1)		
Occupation	Farmer	82/91 (90.1)	1.37	0.71
	Housewife	75/84 (89.3)		
	Employer	41/46 (89.1)		
	Merchant	29/33 (87.9)		
	Other	63/72 (87.5)		
Type of visit	New	187/214 (87.4)	2.10	0.16
	Repeat	103/112 (92.0)		
Payment mode	Cash	157/178 (88.2)	0.46	0.50
	Credit	133/148 (89.9)		

* Statistically significant at *p* < 0.05.

In contrast, no statistically significant associations were observed for other sociodemographic and service‐related variables, including sex (*χ* ^2^ = 0.12, *p* = 0.73), age group (*χ* ^2^ = 0.43, *p* = 0.81), marital status (*χ* ^2^ = 0.02, *p* = 0.88), education level (*χ* ^2^ = 5.12, *p* = 0.08), occupation (*χ* ^2^ = 1.37, *p* = 0.71), visit type (*χ* ^2^ = 2.01, *p* = 0.16), and payment mode (*χ* ^2^ = 0.46, *p* = 0.50). Although satisfaction levels were generally high across these categories, the differences observed were not statistically significant. Overall, these findings indicate that patient satisfaction with pharmacy services was largely consistent across most demographic groups, with residence being the only factor significantly associated with satisfaction in the present study.

## 4 Discussion

This study evaluated patient satisfaction with pharmaceutical services under the APTS system at Bule Hora University Teaching Hospital (BHUTH), providing actionable insights into service quality in a resource‐limited setting. The overall satisfaction rate of 89.0% (95% CI: 85.8–92.2) indicates that APTS implementation has positively influenced patient perceptions of pharmacy services. These findings are consistent with studies in Ethiopian public hospitals [4, 12, 13], which reported that APTS implementation improves transparency, efficiency, and accountability, leading to enhanced patient satisfaction.

The findings of this study indicate high levels of patient satisfaction across several domains of pharmacy service delivery, particularly with respect to the physical environment, pharmacist–patient interaction, and service efficiency. However, relatively lower satisfaction was observed in medication counseling and medicine access, suggesting areas that require further improvement to optimize pharmaceutical care.

Satisfaction with the physical environment was notably high. Patients reported satisfaction with the pharmacy's location (90.5%), cleanliness (96.3%), and counter convenience (98.2%). These findings are consistent with the objectives of the APTS system, which aims to improve the organization, transparency, and physical layout of pharmacy services to enhance patient access and service efficiency. Previous studies conducted in Ethiopian healthcare facilities have reported similar improvements in patient satisfaction following APTS implementation, particularly in relation to pharmacy infrastructure and workflow organization [12, 14, 15, 16]. A well‐organized pharmacy environment contributes not only to improved operational efficiency but also to better patient perceptions of service quality and accessibility.

While clarity of medication instructions achieved relatively moderate satisfaction (85.6%), satisfaction regarding information on storage (70.6%), treatment expectations (74.5%), and side effects (69.3%) was lower. This mirrors a common challenge in developing countries, where pharmacists often focus on dispensing functions and neglect detailed counseling [17]. Inadequate counseling may negatively affect medication adherence and treatment outcomes [18, 19, 20]. Previous studies in Ethiopia reported similar findings: patients were often not informed about storage conditions, side effects, and treatment expectations, which contributed to lower satisfaction scores [15, 16, 21]. Proper medication counseling is essential for adherence and avoiding therapeutic failures, especially in chronic disease management [22]. Previous studies have reported that limited counseling time, high patient load, and insufficient staffing may reduce the quality and depth of medication counseling provided in busy healthcare settings [15, 23]. Adequate medication counseling is essential for promoting safe and effective medication use. Improving pharmacist training in patient counseling and implementing standardized counseling procedures may therefore enhance patient understanding of medications and improve treatment outcomes.

Patients expressed high satisfaction regarding interpersonal skills, including listening to patient concerns (84.4%), time spent with patients (95.1%), courtesy and respect (97.5%), and privacy during consultations (95.4%). This aligns with standard patient satisfaction literature, where consultation time is considered an interpersonal or patient‐centered care measure, not merely an efficiency metric [15, 18]. Respectful and patient‐centered communication is a key determinant of overall satisfaction and trust in healthcare providers [18, 24]. These findings suggest that pharmacists at the study site maintain strong interpersonal and professional communication skills, enhancing the quality of care.

Satisfaction with service efficiency indicators, including promptness (97.2%) and waiting time (96.3%), was very high. These findings are consistent with previous evaluations of APTS implementation, which reported improvements in dispensing efficiency and reductions in waiting time following the introduction of standardized pharmaceutical service procedures [14]. Efficient service delivery is crucial in shaping patient satisfaction, as long waiting times have been consistently reported as a major source of dissatisfaction in healthcare facilities [25]. Efficient pharmacy services contribute significantly to patient satisfaction and improve the overall patient experience within healthcare facilities.

In the medicine access domain, satisfaction with the fairness of cost (98.5%) was high, whereas satisfaction with availability of prescribed medicines (73.0%) was comparatively lower. Studies conducted in Ethiopia and other low‐ and middle‐income countries have shown that medicine stock‐outs and supply chain challenges can negatively affect patient satisfaction and healthcare utilization [18, 24, 26]. Limited availability forces patients to seek medicines from private outlets, increasing costs and risking suboptimal treatments [27]. Availability of prescribed medicines is a critical factor influencing patient satisfaction and adherence to treatment. Strengthening pharmaceutical supply management systems and ensuring consistent availability of essential medicines are therefore essential components of effective pharmaceutical service delivery.

This study also examined the association between selected patient characteristics and overall satisfaction with pharmacy services. The results indicated that place of residence was the only factor significantly associated with patient satisfaction, with urban residents reporting higher satisfaction compared with rural residents. Specifically, 95.7% of urban patients were satisfied with pharmacy services compared with 85.3% of rural patients, and this difference was statistically significant. This finding suggests that geographical differences may influence patient perceptions of healthcare services. Urban residents may have greater exposure to healthcare systems, better access to health information, and higher familiarity with pharmaceutical services, which may positively influence their expectations and experiences. Similar findings have been reported in studies conducted in other healthcare settings where urban patients demonstrated higher satisfaction levels due to better access to healthcare infrastructure and information resources [15, 28].

### 4.1 Strengths and Limitations

Strengths of this study include a sufficiently powered sample, use of a validated multi‐domain questionnaire, and inclusion of both descriptive and inferential statistics. Limitations include the cross‐sectional design, which prevents causal inference, potential social desirability bias inherent in interviewer‐administered surveys, and also the possibility of periodicity bias and reduced randomness if patient flow follows hidden patterns within the sampling frame.

## 5 Conclusion

The implementation of APTS at BHUTH resulted in high overall patient satisfaction. Nevertheless, improvements are still needed in medication counseling practices and medicine supply management. Strengthening pharmacist training, improving counseling practices, and enhancing pharmaceutical supply chain systems may further improve patient satisfaction and the quality of pharmacy services. These measures are essential to maximize the clinical and therapeutic impact of APTS, particularly in resource‐limited rural hospitals.

## 6 Recommendations

Based on the study findings, the following recommendations are proposed:

1. Enhance pharmacist counseling skills:
  Continuous professional development should be provided to pharmacists focusing on communication, patient education, and counseling on medication use, side effects, and storage.
2. Strengthen pharmaceutical supply chain management:
  Addressing medicine availability through improved forecasting, procurement, and stock management practices is critical to reduce frequent medicine stock‐outs.
3. Supervisory and monitoring support:
  Hospital administrators and regulatory bodies should conduct regular audits and supportive supervision to ensure APTS standards are being implemented consistently.
4. Increase public awareness and feedback mechanisms:
  Patients should be educated about their rights to pharmaceutical information, and feedback systems should be established to monitor satisfaction and improve responsiveness.
5. Expand study to other settings:
  Similar studies should be conducted across other health facilities in Ethiopia to assess the nationwide implementation and impact of APTS, ensuring equitable and quality pharmaceutical services.

## Author Contributions

**Aliyi Gerina:** conceptualization, methodology, software, data curation, investigation, validation, formal analysis, supervision, visualization, project administration, resources, writing – original draft, writing – review and editing. **Abduba Wariyo Guyo:** writing – review and editing, visualization, supervision, data curation, and validation. **Woyesa Elema:** investigation, visualization, supervision, and validation.

## Funding

The authors have nothing to report.

## Ethics Statement

This study was reviewed and approved by the Institutional Review Board (IRB) of Bule Hora University, Institute of Health (Ref. No: BHU/IoH/IRB/0426/075/2023). Written informed consent was obtained from all participants prior to data collection. All study procedures adhered to the ethical principles outlined in the Declaration of Helsinki.

## Consent

Participants were fully informed about the study's purpose, procedures, confidentiality safeguards, and their right to withdraw at any time without penalty.

## Conflicts of Interest

The authors declare no conflicts of interest.

## Transparency Statement

The lead author, Aliyi Gerina, affirms that this manuscript is an honest, accurate, and transparent account of the study; no important aspects have been omitted; and any deviations from the protocol are explained.

## Data Availability

The data sets generated and analyzed during this study are available from the corresponding author upon reasonable request.
